# Clickotine, A Personalized Smartphone App for Smoking Cessation: Initial Evaluation

**DOI:** 10.2196/mhealth.7226

**Published:** 2017-04-25

**Authors:** Brian M Iacoviello, Joshua R Steinerman, David B Klein, Theodore L Silver, Adam G Berger, Sean X Luo, Nicholas J Schork

**Affiliations:** ^1^ Click Therapeutics, Inc. New York, NY United States; ^2^ Department of Psychiatry Icahn School of Medicine at Mount Sinai New York, NY United States; ^3^ The Translational Genomics Research Institute Phoenix, AZ United States

**Keywords:** smoking cessation, cigarette smoking, tobacco, therapeutics, smartphone

## Abstract

**Background:**

Tobacco smoking is the leading cause of preventable death in the United States, and the annual economic burden attributable to smoking exceeds US $300 billion. Obstacles to smoking cessation include limited access and adherence to effective cessation interventions. Technology can help overcome these obstacles; many smartphone apps have been developed to aid smoking cessation, but few that conform to the US clinical practice guideline (USCPG) have been rigorously tested and reported in the literature. Clickotine is a novel smartphone app for smoking cessation, designed to deliver the essential features of the USCPG and engineered to engage smokers by personalizing intervention components.

**Objective:**

Our objective was to assess the engagement, efficacy, and safety of Clickotine in an initial, single-arm study. Outcomes measured were indicators of engagement with the smartphone app (number of app opens, number of interactions with the Clickotine program, and weeks active with Clickotine), cessation outcomes of 7- and 30-day self-reported abstinence from smoking, and negative health events.

**Methods:**

We recruited US residents between 18 and 65 years of age who owned an iPhone and smoked 5 or more cigarettes daily for the study via online advertising. Respondents were prescreened for eligibility by telephone and, if appropriate, directed to a Web portal to provide informed consent, confirm eligibility, and download the Clickotine app. Participants completed study assessments via the online portal at baseline and after 8 weeks. Data were collected in Amazon S3 with no manual data entry, and access to all data was maximally restrictive, logged, and auditable.

**Results:**

A total of 416 participants downloaded the app and constituted the intention-to-treat (ITT) sample. On average, participants opened the Clickotine app 100.6 times during the 8-week study (median 69), logged 214.4 interactions with the Clickotine program (median 178), and remained engaged with Clickotine for 5.3 weeks (median 5). Among the ITT sample, 45.2% (188/416) reported 7-day abstinence and 26.2% (109/416) reported 30-day abstinence from smoking after 8 weeks. Completer analysis focused on 365 (87.7%) of the 416 enrolled participants who completed the 8-week questionnaire revealed that 51.5% (188/365) of completers reported 7-day abstinence and 29.9% (109/365) reported 30-day abstinence. Few adverse events, mostly consistent with nicotine withdrawal symptoms, were reported and overall no safety signal was detected.

**Conclusions:**

In this initial single-arm trial, Clickotine users appeared to demonstrate encouraging indicators of engagement in terms of the number of app opens, number of program interactions, and continued engagement over time. Clickotine users reported encouraging quit rates while reporting few adverse events. Future research is warranted to assess Clickotine’s efficacy in a randomized controlled trial.

**Trial Registration:**

Clinicaltrials.gov NCT02656745; https://clinicaltrials.gov/ct2/show/NCT02656745 (Archived by WebCite at http://www.webcitation.org/6peTT4x60)

## Introduction

The burden of mortality and disease from tobacco use in the United States is extensive. According to the latest report of the Surgeon General regarding the consequences of 50 years of tobacco use in the United States, tobacco smoking is the leading cause of preventable death [1]. Each year the numbers surpass 480,000 deaths; 16 million people live with diseases brought on by smoking; secondhand smoke contributes to the death of more than 41,000 others annually; and approximately 5.6 million children alive today who are younger than 18 years will die prematurely as a result of smoking [2]. It is estimated that 15% of all Americans aged 18 or older smoke tobacco in some form [3], with 9 out of 10 adult cigarette smokers developing a tobacco use disorder before their 18th birthday [4]. Annually, the total economic cost attributable to smoking is now over US $300 billion, with US $170 billion in direct medical costs [5] and US $156 billion in productivity losses [2].

In 2010, approximately 7 out of 10 adult cigarette smokers expressed a desire to quit [6]. However, most quit attempts fail: only 3% to 5% of smokers maintain abstinence up to 1 year after quitting [7]. On the other hand, dissemination and accessibility of proven interventions continue to be limited. For instance, cessation therapies are not always widely accessible and, if they are, they tend to serve only a small population of heavy smokers [7,8]. Furthermore, about 25% of patients looking to stop smoking do not take prescribed medicine as directed [9].

A solution to overcome these obstacles is to develop cessation programs that include effective quit plans with substantial population-level impact and that better ensure long-term adherence. New technologies constitute a promising opportunity to do this at the lowest cost due to high population reach and immediate accessibility [10,11]. A large assessment study in a US cohort found that 76% of smokers own smartphones, meaning smoking cessation apps could be accessible for most smokers and are a worthwhile option to consider [12]. Consequently, in recent years, there has been a proliferation of apps designed to help users quit smoking. In 2013, there were 400 smoking cessation smartphone apps available in the United States, with over 3.2 million downloads in the United States alone [13], but only a small number of these apps [14] appear to follow the US clinical practice guideline (USCPG) on treating tobacco use and dependence [15]. Although the effectiveness of many available smartphone apps for smoking cessation does not appear to have been rigorously tested or reported, efficacy studies of several recently developed apps have been published (for example, apps that deliver Acceptance and Commitment Therapy components [16], or text message-based apps that provide support and interaction [17]). However, a recent review of the content included in smartphone apps for smoking cessation revealed that most of those evaluated did not include behavior change techniques that have been shown to be effective in smoking cessation interventions [18], and most available apps are not customized to users’ needs or personal characteristics [19]. Thus, the development of smartphone apps to deliver smoking cessation interventions appears to be warranted, given the demonstrated need for such tools and early evidence that these apps can be efficacious if they are designed to include empirically supported behavior change techniques.

Clickotine is a novel smartphone app, designed and engineered to deliver essential features of the USCPG that are amenable to delivery via an app (advise and encourage to quit; assess willingness to quit and enhance motivation; assist with quit planning and connect with intervention, including advice on pharmacotherapy, connection with counseling and medication treatments, provision of social support, and connection with a quitline; and arrange or provide follow-up) [15]. Through a series of missions and interactions with the app, Clickotine delivers these features, as well as empirically supported smoking cessation intervention components (see App Description section below for a description of the Clickotine program and empirical support). The USCPG also recommends personalizing these features as much as possible to maximize their efficacy. An adaptive proprietary technology platform, Clickometrics, was engineered to enhance engagement by personalizing the smoking cessation intervention components that are delivered. This innovative program is hypothesized to help individuals quit smoking safely and effectively.

In this paper, we report initial results of an 8-week single-arm clinical trial, in which we enrolled 416 participants to assess the engagement and efficacy of Clickotine. Engagement with an app is important to evaluate in preliminary studies, as these interventions will only exert an effect if the users actually use them. High attrition rates are often observed for mobile health apps, potentially limiting their effectiveness [20,21], and discontinuation of smartphone app use is a problem: approximately 26% of app users discontinue after one use, and 74% discontinue by the 10th use [22]. Adherence to smartphone interventions has been shown to predict smoking cessation [23]. It is therefore important to evaluate the engagement with novel smartphone cessation apps in preliminary studies along with efficacy. Indicators of engagement measured in this study were the number of app opens, the number of interactions with the Clickotine program components, and the number of weeks that users remained active with the Clickotine program. In addition to engagement, we also measured preliminary indicators of efficacy, namely 7- and 30-day self-reported point prevalence of abstinence from smoking, after 8 weeks.

## Methods

We conducted an 8-week, single-arm clinical trial of Clickotine (Click Therapeutics, Inc, New York, NY, USA). All study procedures were reviewed and approved by Western Institutional Review Board (IRB) (Puyallup, WA, USA). The trial was registered with clinicaltrials.gov (NCT02656745).

### Participants

To be eligible, participants had to be aged 18 to 65 years, smoke at least five cigarettes daily, want to quit smoking in the next 30 days, own an iPhone with iOS 8 or higher capabilities, be willing and able to receive text messages, be able to comprehend the English language, live in the United States, and provide informed consent. The study aimed to include participants who were current daily smokers. We chose the daily cigarette cutoff of 5 to be consistent with other studies of comparable apps (eg, Bricker et al [16]). We also included desire to quit smoking as an inclusion criterion based on other cessation app studies’ inclusion criteria (eg, Bricker et al [16]). However, we assumed desire to quit based on participants responding to the digital advertisement, and we asked them about it during the phone screening call although we did not formally measure it.

### Recruitment

We recruited potential participants from May to July 2016. Digital advertisements were posted to social media outlets (Facebook, Craigslist, Twitter, Instagram, and Reddit), targeting users who searched for “quit smoking” where possible. Digital advertisements contained IRB-approved copy: “Ready to quit? We’re ready to help. If you have an iPhone and want to become smokefree while earning up to $100, we may have a great solution for you.” The study director or a study team member under his supervision contacted respondents by telephone to prescreen for eligibility and, if appropriate, directed them to a Web portal for the study. After providing online informed consent and confirming eligibility, participants were sent an email with a secure link to download the app. Providing informed consent and downloading the app constituted enrollment in the study; these respondents constituted the intention-to-treat (ITT) sample for the study.

### App Description

Clickotine adheres to the USCPG essential content features for smoking cessation. The USCPG was developed for in-person clinical settings and, as such, not all parts of the guideline will apply for mobile phone apps [14]. In designing the Clickotine program, we followed the parts of the guideline that are appropriate and amenable for inclusion in a digital therapeutic application: advise and encourage to quit; assess willingness to quit and enhance motivation; assist with quit planning and connect with intervention, including counseling and medications, advice on pharmacotherapy, provision of social support, and connection with a quitline; and arrange or provide follow-up. These USCPG features were developed in consideration of empirical support for their efficacy. For advising and encouraging to quit, evidence exists that even brief advice to quit from a counselor or health care provider significantly increases long-term smoking abstinence rates [24]. Support for assessing willingness to quit and enhancing motivation exists, as evidence suggests that a variety of motivational interventions can increase motivation for behavior change, including smoking cessation [25-27]. The effectiveness of encouragement and support as part of smoking cessation treatment (assisting with quit planning and connecting with appropriate interventions) is consistent with the literature regarding the importance of providing a caring, empathic, and understanding context in making health behavior changes [28-30]. Evidence for the effectiveness of arranging and providing follow-up in smoking cessation treatment also exists—the USCPG recommends that assessments within the first week after quitting should be encouraged, as these can minimize relapse in quitters or encourage abstinence in prequitters [31,32].

**Figure 1:**
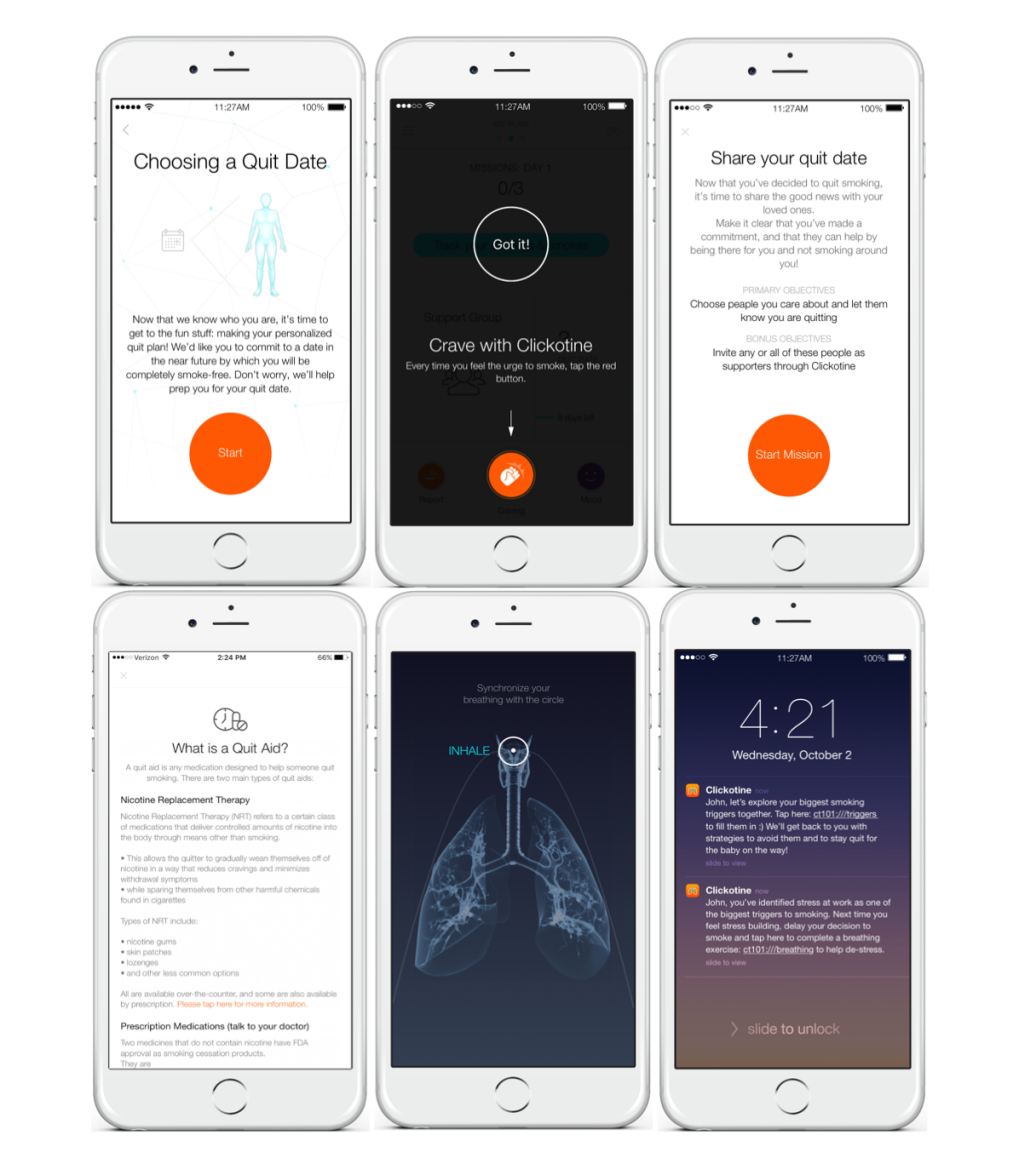
Examples of interactions with the Clickotine program (left to right, beginning at top): choose a quit date; log cravings; mission: share quit date with supporters; learn about quit aids; control breathing; receive a personalized message.

Clickotine follows these important USCPG guidelines and personalizes these as much as possible via the Clickometrics platform, which is engineered to enhance engagement. Upon downloading the Clickotine app to their iPhone, users are prompted to create a user profile and answer a brief questionnaire on smoking behavior. The users then self-select a quit date between 7 and 21 days after creating their user profile. Based on the chosen quit date, personal characteristics, and smoking characteristics (eg, name, age, sex, location, quit motives, smoking history, and even the desired number of messages per day as indicated by the user), users receive a tailored plan of missions and messages. Within the context of the USCPG features identified above, Clickotine delivers intervention components that have demonstrated efficacy in promoting smoking cessation: controlled breathing [33], personalized messaging [34,35], social engagement [36], encouragement of pharmacotherapy for cessation and of medication adherence [37,38], and digital diversions, including targeted strategies to cope with cravings, withdrawal symptoms, and lapses (such as reviewing positive moments or previous successes in weathering cravings) [39]. Delivery of these components in adherence to the USCPG results in a set of interactions for the user: controlling breathing, using digital diversions, logging cravings, receiving personalized messages, responding to messages, participating with “quit teams,” completing missions, logging sentiments and feelings, logging cigarettes smoked, journaling, learning about and using quit aids, and interacting with supporters linked through the app. Table 1 demonstrates the relationship between the USCPG features and the set of interactions experienced by the Clickotine user, and Figure 1 provides representative screenshots of Clickotine interactions. The Clickotine interaction categories do not map 1-to-1 onto the USCPG features, and some interaction categories apply to multiple USCPG features or stages of the Clickotine program. For example, some Clickotine missions are designed to advise and encourage quitting (eg, learn about the health effects of smoking), while others are relevant for assessing willingness and enhancing motivation to quit (eg, exploring the user’s quit motives); interacting with supporters is relevant for enhancing motivation to quit in the early stages (eg, connecting with supporters in-app, sharing your desire to quit, and asking for support), as well as being part of cessation intervention in the later stage of the program (eg, updating supporters on progress); and logging cravings or cigarettes smoked can be a mechanism for enhancing motivation to quit and could also be a component of effective intervention. Over the course of a user’s quit journey, in-app interactions are emphasized until the quit date, whereas personalized messaging is designed to become the primary engagement modality following the quit date. In general, in this study, we sent a minimum of 1 personalized message per day to participants as long as they had not deleted the app. If participants responded to a personalized message or completed a mission in response, they were more likely to receive another; thus, participants received different numbers of personalized messages.

**Table 1:** US clinical practice guideline (USCPG) features and associated Clickotine interaction categories.

Guideline	Clickotine interaction category
Advise and encourage to quit	Complete missions
Assess willingness to quit and enhance motivation	Complete missions Log sentiments and feelings Participate with “quit teams” Interact with supporters Log cravings Log smokes
Assist with quit planning and connect with intervention	Complete missions Control breathing Use digital diversions Participate with “quit teams” Interact with supporters Write in a journal Learn about and use quit aid Receive personalized messages Respond to messages Log cravings Log smokes
Arrange or provide follow-up	Receive personalized messages Respond to messages Log cravings Log smokes Log sentiments and feelings

### Data Collection

We collected baseline demographic and smoking characteristic data via an online survey for participants who met the eligibility criteria and gave informed consent. We also collected data on smoking behavior, as well as engagement metrics including the number of app opens and interactions with the Clickotine program components in-app after downloading. We administered a Web-based outcome survey 8 weeks after enrollment and, in return for completing the 8-week survey, provided participants with a US $25 Amazon gift certificate. Participants were notified to take the online survey via text message with a link to the survey on day 53 (3 days before reaching 8 weeks after initial consent). Each day thereafter until participants completed the survey, up to 7 days after the target date, they were contacted via text message, email, or phone call. A sample text message is: “Good Morning [name]! Your 8-week survey is ready! Fill it out online to get your first Amazon gift card: http://xxxx.” These in-app and online survey data were collected in Amazon Simple Storage Service (Amazon S3; Amazon Web Services, Seattle, WA, USA) with no manual data entry, and access to all data was maximally restrictive, logged, and auditable.

A priori, the study data management plan defined 3 categories of study team members: (1) participant-facing (having some interaction with participants; eg, recruitment or in-app), (2) data team (maintaining the study database), and (3) study leadership (not interacting directly with participants, and not having access to the study database; only conducting analyses on relevant variables provided by the data team after the database lock). This means that access to data was restricted only to the minimum set of actors in the minimal set of use cases needed to complete the study. As per the approved study protocol, no one outside of the designated data team had access to the data and even the data team could not modify it. This procedure follows the principle of least privilege [40]. This was just one of the steps taken to ensure maximal objectivity and transparency in this study conducted by the sponsor. Moreover, the process that wrote the data from users to the database was restricted to write only those files that were relevant to it and had no ability to read data. All app and survey data were stored in an isolated network. All survey data were collected via preprogrammed, automated processes, and all collected data were immediately made immutable and could not be changed by the sponsor. These procedures were approved by an independent IRB and were designed and implemented to minimize potential biases unconsciously introduced by the sponsor, which might have influenced the study data.

### Measurements

#### Baseline Characteristics

We ascertained baseline demographic characteristics (age, sex, race/ethnicity) and smoking characteristics (years smoking, number of cigarettes smoked per day) via questions in the online survey. Baseline nicotine dependence level was measured using the Fagerstrӧm Test for Nicotine Dependence [41], administered in the online survey upon study enrollment.

#### Engagement

We measured engagement with the smartphone app in three ways: the number of app opens; the number of interactions with program components beyond app opens (see App Description above); and the number of weeks in which the user remained actively engaged with Clickotine (defined as having at least one interaction with the program in the week, other than passive receipt of a message). App opens is a standard and important metric for measuring engagement with a digital therapeutic, for smoking cessation [42] or other indications. App opens could be tallied over the course of the entire 8-week study. Clickotine interactions could also be tallied over the entire course of the study, across the categories in total and by category (controlling breathing, using digital diversions, logging cravings, receiving personalized messages, responding to messages, participating with “quit teams,” completing missions, logging sentiments and feelings, logging cigarettes smoked, journaling, learning about and using quit aids, and interacting with supporters linked through the app). It was not possible to confirm that a user read a personalized message, so the count for “receiving personalized messages” is the number of personalized messages the user was sent.

#### Smoking Cessation

To detect a signal for smoking cessation in this short-term trial, the 8-week questionnaire assessed self-reported 7-day and 30-day point prevalence abstinence [43-45]. Self-reported smoking is a standard method for assessing the efficacy of low-intensity interventions [46,47], which we used in this study. Users answered the following question for 7- and 30-day time frames: “Have you smoked (even a puff) in the last [7 or 30] days?”

#### Medical Monitoring

Negative health events were ascertained via spontaneous report by users in-app and via proactive ascertainment by a focused question in the week-8 questionnaire: “At any point during the study did you experience a negative health event?” All negative health events reported were sent to the medical monitor for evaluation. The medical monitor assessed each event and, when needed in order to comprehensively assess and document reported events, requested additional information from study team members in contact with the participant. All events judged to be clinically significant by the medical monitor were considered to be adverse events. The medical monitor also determined relatedness to Clickotine (possibly related, not related) based on all available information and clinical judgment. The intervention confers minimal risk and, other than referral when appropriate, we delivered no medical intervention.

### Data Analysis

We calculated descriptive statistics to estimate engagement with the app and smoking cessation rates. Engagement was quantified in three ways: the number of app opens during the 8-week study, the number of Clickotine program interactions experienced by the user (in total, and for each interaction category), and the number of weeks the user remained actively engaged with Clickotine. We estimated population-level engagement as the mean (SD) values across participants or median (interquartile range) values for instances of nonnormally distributed variables. Smoking abstinence rates were calculated as the proportion of the ITT sample that self-reported not smoking, not even a puff, for at least 7 days and at least 30 days at the 8-week study end point. In this ITT analysis, nonresponders to the 8-week survey were imputed as smokers. This method has been shown to yield potentially biased results, by potentially inflating the proportion of smokers compared with a completer analysis [48]. Despite the potential for bias in smoking cessation trials, this method for ITT analysis is expected for traditional clinical trials and was conducted in this study. However, we also conducted completer analyses on the sample that completed the 8-week outcome survey for comparison with the ITT results.

We conducted post hoc analyses using logistic regression, due to the nonnormal distributions of most of the predictor variables and the dichotomous outcome variables, to explore associations between baseline characteristics and engagement indicators, between baseline characteristics and cessation outcomes, and between engagement indicators and cessation outcomes in this sample. As these were exploratory, hypothesis-generating analyses, we evaluated each independent variable (baseline sex, age, race/ethnicity, nicotine dependence level, years smoked, cigarettes per day, and number of previous quit attempts) as a predictor in a separate model, with app opens, Clickotine interactions, and number of weeks active dichotomized into high/low groups according to median split. We corrected critical *P* values for multiple comparisons familywise (eg, .05/7=.007 for the analyses of 7 baseline predictors of each engagement indicator). Similar models were run with 7-day and 30-day cessation as the binary outcome to explore associations between baseline characteristics and outcomes, with the same *P* value correction (.05/7). Logistic regression analyses were also conducted with app opens, Clickotine interaction counts, and active week counts in separate models as engagement predictors of cessation outcomes, with *P* values corrected for 3 comparisons for each outcome (.05/3=.02). Visual inspection and analyses were conducted using IBM SPSS Statistics for Mac version 24.0 software (IBM Corporation).

## Results

### Recruitment and Study Enrollment

**Figure 2:**
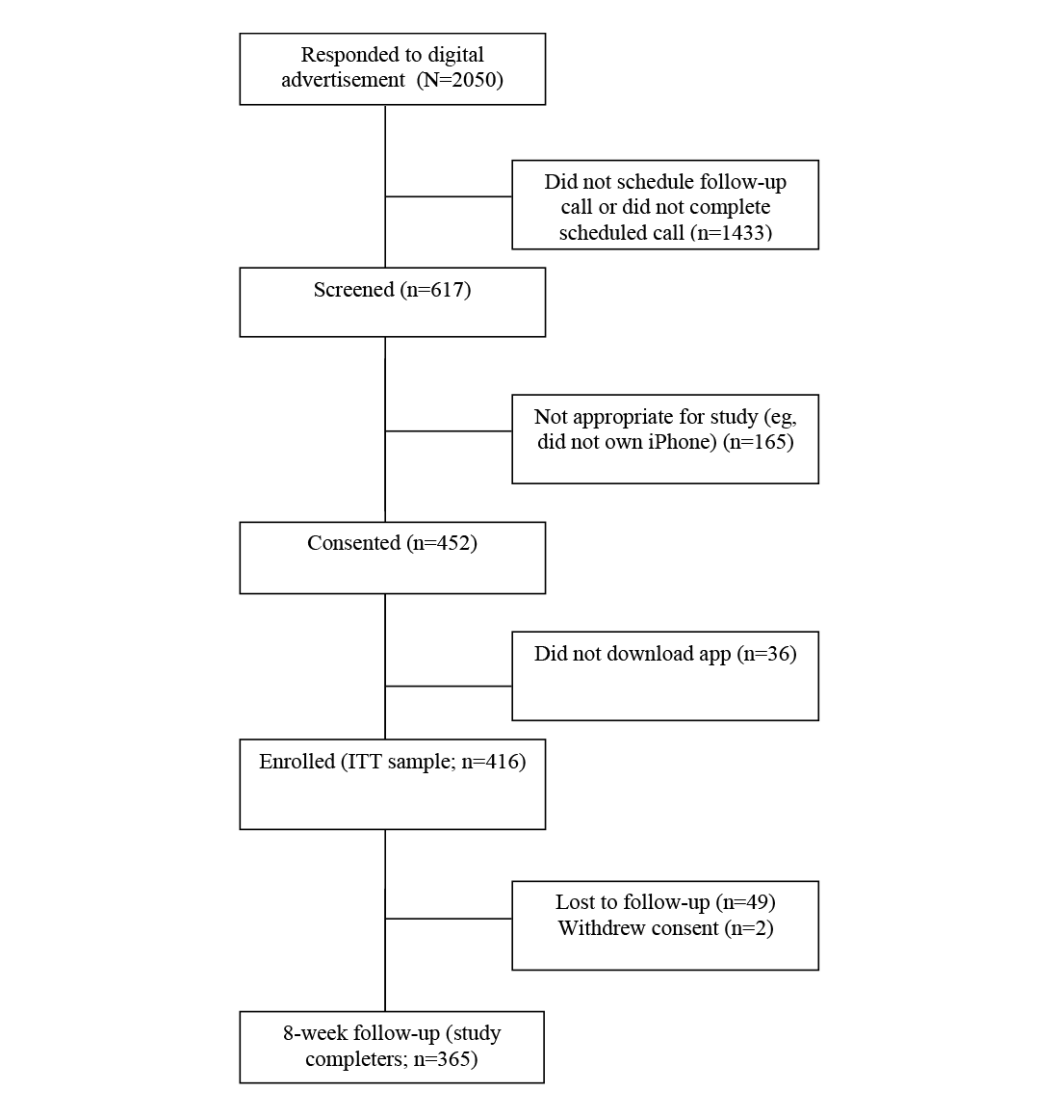
Study flow diagram. ITT: intention-to-treat.

After 63 days of social media advertising and recruitment, we received 2050 contacts and conducted 617 telephone prescreens of potentially eligible participants. Many of the 2050 respondents did not schedule or respond to the prescreening call. Screening calls were conducted with participants until approximately 600 were completed, which was the target to yield a targeted ITT sample of >400. This resulted in 452 participants invited to provide online informed consent and who were emailed a secure link to download the app. Of these, 416 participants ultimately downloaded the app and constituted the ITT population. Of the 416 participants in the ITT sample, 365 completed the 8-week outcome questionnaire, yielding an 87.7% retention rate. Figure 2 depicts the study flow diagram for this trial.

Table 2 provides the demographic and smoking characteristics of the study sample. At baseline, this sample reported a high degree of nicotine dependence: the mean score on the Fagerstrӧm Test for Nicotine Dependence was 6.1 (SD 2.2), which falls in the high range for nicotine dependence (scores between 6 and 7) [49]. On average, participants had been smoking for 18.1 (SD 10.6) years, and were smoking 16.7 (SD 7.8) cigarettes per day. At baseline, 29 participants (7.0% of the ITT sample) were using a pharmacotherapy smoking cessation aid (nicotine replacement therapy or medication). At study outcome, 66 participants (15.9% of the ITT sample) were using a pharmacotherapy smoking cessation aid (nicotine replacement therapy or medication).

### Engagement Indicators

The distributions were significantly nonnormal for app opens (*W*_416_=.773, *P*<.001), Clickotine program interactions (*W*_416_=.809, *P*<.001), and the number of active weeks with Clickotine (*W*_416_=.892, *P*<.001) according to Shapiro-Wilk tests of departure from normality. Table 3 provides the measures of central tendency of these data: mean (SD), range, median, and interquartile range. Table 4 describes the frequency with which users encountered each interaction category, as well as the proportion of program interactions each category represented.

**Table 2:** Demographic and smoking characteristics of the study sample.

Characteristics			Mean (SD) or n (%)
**Demographics**			
	Age in years, mean (SD)		36 (10.8)
	Female, n (%)		247 (59.4)
	**Race/ethnicity, n (%)**		
		White	315 (75.7)
		Hispanic	37 (8.9)
		African American	22 (5.3)
		Asian or Pacific Islander	11 (2.6)
		Native American	5 (1.2)
		Other/no response	26 (6.3)
**Smoking characteristics**			
	Fagerstrӧm score, mean (SD)		6.1 (2.2)
	No. years smoking, mean (SD)		18.1 (10.6)
	No. cigarettes per day, mean (SD)		16.7 (7.8)
	**No. of previous quit attempts in past year, n (%)**		
		0	88 (21.2)
		1	119 (28.6)
		>1	166 (39.9)
		Did not respond	43 (10.3)

**Table 3:** Engagement indicators over the 8-week study period.

Indicator	Mean (SD)	Median	Range	Interquartile range
App opens	100.6 (98.2)	69.0	3-780	36.0-134.75
Clickotine program interactions	214.4 (158.4)	178.0	20-1213	110.25-273.0
Weeks active with Clickotine	5.3 (2.4)	5.0	0-8	3.0-8.0

**Table 4:** Clickotine interactions by category over the 8-week study period.

Interaction category	Mean (SD)	% of total interactions	Median	Range	Interquartile range
Receiving personalized messages	84.06 (44.87)	39.2	79.0	1-372	59-94.75
Logging cigarettes smoked	53.61 (73.78)	25.0	33.0	0-964	12-73
Completing missions	27.83 (22.48)	13.0	23.5	0-114	9-39
Logging cravings	15.09 (26.89)	7.0	5.0	0-224	2-15
Responding to messages	10.16 (24.12)	4.7	3.0	0-337	1-11
Controlling breathing	6.94 (10.49)	3.2	4.0	0-105	2-8
Journaling	4.42 (7.78)	2.1	2.0	0-87	0-5
Participating with quit teams	3.52 (15.21)	1.6	0.0	0-171	0-0
Logging sentiments and feelings	2.66 (6.69)	1.2	0.0	0-76	0-2
Using digital diversions	2.54 (11.90)	1.2	0.0	0-211	0-1
Learning about and using quit aid	1.82 (10.16)	0.9	0.0	0-165	0-1
Interacting with supporters	1.74 (4.04)	0.8	0.0	0-53	0-2
Total	214.4 (158.4)				

### Cessation Outcomes

Table 5 summarizes the results of the ITT and completer analyses of cessation outcomes. In the ITT sample (n=416), at the end of the 8-week study period, 45.2% (n=188) of participants reported achieving 7-day abstinence and 26.2% (n=109) of participants achieved 30-day abstinence. Among the 365 study completers, 51.5% (n=188) of participants reported achieving 7-day abstinence and 29.9% (n=109) of participants achieved 30-day abstinence. In this study, participants did not appear to reliably provide the in-app weekly smoking data. Only 47 of the 416 participants (11.3%) provided complete data in the weekly cigarette counts; across all users, only 822 of the 3328 (24.7%) weekly cigarette counts were completed. Thus, we derived smoking status from self-report in the baseline and outcome surveys, and we did not use the in-app smoking data in the analyses of cessation outcomes.

### Safety Outcomes

Participants reported 4 negative health events spontaneously, and reported an additional 32 in response to the focused safety question in the 8-week questionnaire. Of these 36 negative health events, 19 were considered clinically significant and documented as adverse events. Of the 19 adverse events, 2 were considered possibly related to Clickotine use in the judgment of the medical monitor (nightmare, depressed mood). The most common adverse events were fatigue (reported by 3 participants) and mood change (reported by 2 participants); no additional adverse events occurred in more than 1 participant.

### Associations Between Baseline Characteristics, Engagement Indicators, and Cessation Outcomes

Table 6 provides the results of the logistic regression analyses of baseline characteristics predicting engagement indicators. The only association surviving correction for multiple comparisons was female sex predicting an increased likelihood of being in the high app opens group.

Table 7 provides the results of the logistic regression analyses of baseline characteristics predicting cessation outcomes. Older age and greater number of years smoking appeared to predict a decreased likelihood of reporting 7-day abstinence and 30-day abstinence. Greater number of cigarettes smoked per day predicted a decreased likelihood of reporting 30-day abstinence.

Table 8 provides the results of the logistic regression analyses of engagement indicators predicting cessation outcomes. Greater number of weeks active with Clickotine was associated with an increased likelihood of reporting 7-day and 30-day abstinence.

**Table 5:** Intention-to-treat (ITT) and completer analysis results for smoking cessation.

Duration of abstinence	ITT analysis (n=416), n (%)	Completer analysis (n=365), n (%)
7 days	188 (45.2)	188 (51.5)
30 days	109 (26.2)	109 (29.9)

**Table 6:** Logistic regression analyses of baseline predictors of engagement indicators.

Engagement indicators		Age	Sex	Race/ethnicity	Fagerstrӧm score	Years smoking	Cigarettes per day	Prior quit attempts
**Clickotine app opens (high/low according to median split)**								
	OR^a^	1.018	0.563	1.044	1.071	1.019	1.025	0.990
	95% CI	1.00-1.04	0.38-0.84	0.95-1.15	0.98-1.17	1.00-1.04	0.999-1.05	0.90-1.00
	*P* value	.048	.005^b^	.36	.12	.047	.06	.7
**Clickotine program interactions (high/low according to median split)**								
	OR	1.018	0.668	0.990	1.070	1.023	1.026	0.981
	95% CI	1.00-1.04	0.45-0.99	0.90-1.09	0.98-1.17	1.00-1.04	1.00-1.05	0.89-1.01
	*P* value	.045	.04	.83	.13	.02	.047	.76
**No. weeks active (>0 interactions/week)**								
	OR	0.997	0.690	1.096	0.932	1.001	0.969	1.004
	95% CI	0.98-1.01	0.47-1.02	1.00-1.21	0.86-1.02	0.98-1.02	0.94-1.00	1.00-1.01
	*P* value	.75	.07	.06	.11	.92	.02	.24

^a^OR: odds ratio.

^b^*P*<corrected α (.05/7=.007).

**Table 7:** Logistic regression analyses of baseline predictors of smoking cessation outcomes.

Cessation outcomes		Age	Sex	Race/ ethnicity	Fagerstrӧm score	Years smoking	Cigarettes per day	Prior quit attempts
**7-day cessation**								
	OR^a^	0.969	1.415	1.094	0.948	0.965	0.976	0.987
	95% CI	0.95-0.99	0.95-2.10	1.00-1.20	0.87-1.03	0.95-0.98	0.95-1.00	0.91-1.10
	*P* value	.001^b^	.08	.06	.23	<.001^b^	.06	.74
**30-day cessation**								
	OR	0.964	1.092	1.020	0.945	0.954	0.954	1.034
	95% CI	0.95-0.98	0.70-1.70	0.92-1.13	0.86-1.04	0.93-0.98	0.92-0.99	0.94-1.14
	*P* value	.001^b^	.7	.70	.25	<.001^b^	.005^b^	.37

^a^OR: odds ratio.

^b^*P*<corrected α (.05/7=.007).

**Table 8:** Logistic regression analyses of engagement indicators predicting smoking cessation outcomes.

Cessation outcomes		App opens	Interaction count	Active weeks
**7-day cessation**				
	OR^a^	1.001	1.001	1.218
	95% CI	0.999-1.003	0.999-1.002	1.12-1.33
	*P* value	.27	.25	<.001^b^
**30-day cessation**				
	OR	1.001	1.001	1.283
	95% CI	0.998-1.003	0.999-1.002	1.16-1.42
	*P* value	.57	.31	<.001^b^

^a^OR: odds ratio.

^b^*P*<corrected α (05/3=.017).

## Discussion

In this initial digital study of Clickotine conducted with no in-person visits, we assessed preliminary indicators of the engagement with and efficacy of Clickotine. We measured engagement using three indicators: number of app opens (median 69), number of Clickotine program interactions experienced by the user (median 178), and the duration of active Clickotine use (number of weeks active; median 5). These engagement indicators suggest that participants were actively using Clickotine during the 8-week study period. Clickotine also appeared to be effective for smoking cessation: 26.2% of participants reported achieving 30-day abstinence after 8 weeks. The most commonly reported adverse events, fatigue and mood change, are expected nicotine withdrawal symptoms [50]. Overall, we detected no safety signal in this study, and we expect no adverse reactions with Clickotine use.

The user experience with Clickotine can be described based on the frequency of the various program interactions a user encountered. In this study, the most frequently encountered feature was receiving personalized messages, which accounted for 39.2% of Clickotine interactions on average. Logging cigarettes smoked was the second most frequent category, accounting for 25% of interactions. Completing missions was the third most frequently encountered category, which accounted for 13% of interactions. For 5 of 12 interaction categories, the median frequency was 0, indicating that roughly half of participants did not encounter this type of interaction during their use of Clickotine. This variability in the interaction categories that participants encountered reinforces the personalized and unique journey on which Clickotine takes each individual user.

We conducted post hoc analyses to explore associations between baseline characteristics, engagement indicators, and cessation outcomes, as some associations have been suggested by previous studies. For example, older age and female sex have been associated with increased engagement with Web-based cessation interventions [51] but have not been found to be associated with use of a smartphone-based smoking cessation app [52]. Heavier smoking has been observed to be associated with use of Web-based interventions and smartphone apps [52,53]. Engagement with smartphone cessation apps, also referred to as adherence, has been shown to predict cessation outcomes [23]. Due to the exploratory, post hoc nature of the logistic regression analyses we conducted in this study, we have interpreted the results with caution to identify associations of interest for future study without drawing much of a conclusion about these associations in the context of this study. In these analyses, female sex appeared to be associated with increased in-app opens. Older age, greater number of years smoking, and greater number of cigarettes smoked per day appeared to be associated with a decreased likelihood of smoking cessation. Results suggested a possible association between an increase in the number of weeks active with Clickotine and an increased likelihood of smoking cessation. Not enough research has been published on predictors of smartphone app use to make comparisons with our study results but, in comparison with one recent study [52], some of our results are contradictory: female sex appeared to be associated with increased app opens, whereas in the previous study female sex was associated with lower use of certain app features. However, our study also appeared to show a trend toward heavier smoking being associated with increased app use, which would be consistent with the previous study [52]. Nonetheless, these associations will be investigated further in the next randomized controlled trials of Clickotine that are conducted, in which statistical and clinical inference will be possible.

This study was efficiently designed and conducted. No in-person visits occurred, and all but one study interaction (the prescreening phone call) was conducted electronically or in-app. This study design enabled the study sponsor to handle a large number of responders to the digital advertising (2050 responses) and enroll the target sample size (400) in a relatively short time (63 days). The study retention rates were encouraging: 365 of the 416 participants completed the 8-week outcome questionnaire, yielding an 87.7% retention rate in this trial. This rate was higher than the retention rates observed in other cessation trials. In one review of 28 Web-based studies, only 8 demonstrated retention rates greater than 80%, 8 demonstrated 50% to 80%, and 12 demonstrated retention rates lower than 50% [54]. In another review of 11 studies [55], the average attrition rate at last follow-up was 38.2%. Comparisons with these studies must be made with caution, however, as these were Web based, whereas our study was smartphone based; the follow-up periods were variable across studies, and some were longer than the 8-week period of our study; and we compensated participants for their time in our study, whereas participants did not receive compensation in some of the other studies. These factors could yield an inflated retention rate in our sample compared with other studies. In comparison, a recent smartphone-based app study, which included similar compensation for participation and was also a single-arm study, reported a retention rate of 84.8% [42]; another similar study comparing 2 different apps reported a retention rate of 82.8% [16]. These rates are similar to the rate of 87.7% observed in our study and bolster confidence in this value as representative of a digital trial of a smartphone-based smoking cessation app.

We studied Clickotine in isolation in this trial and did not directly compare it with other app-based interventions. Although there are factors that limit the ability to compare across unrelated trials, such as differences in sample characteristics and trial methodologies, descriptive rates of engagement and smoking cessation can provide preliminary indications of comparability. In this study, the outcomes observed for Clickotine numerically exceed those of the most comparable mobile apps that have been clinically tested, such as SmartQuit 2.0 (16.6 app opens in an 8-week study and 11% 30-day abstinence in a completer analysis) [42] and the National Cancer Institute’s QuitGuide (15.2 app opens in an 8-week study and 8% 30-day abstinence in ITT analysis) [16]. We propose that components unique to Clickotine may contribute to enhanced engagement and efficacy, including Clickotine’s highly personalized features.

Certain limitations of this study warrant further discussion. First, the follow-up period was relatively short; substantial relapse naturally occurs after a 2-month follow-up [56] and only about 3% to 5% of smokers maintain abstinence up to 1 year after quitting [7]. We will continue to observe participants in this study and will report longer-term outcomes separately when available. Nonetheless, this study was consistent with previous smoking cessation studies in reporting 30-day point prevalence abstinence rates. Second, we tested only the iPhone version of the app in this study, as the Android version was not yet fully developed by the time of study launch. Also, the average age of participants in this study (36 years) is younger than is typical for smoking cessation trials. Inclusion of younger, iPhone-only users may limit the generalizability of findings. For example, one cessation app study noted that iOS users demonstrated indicators of greater motivation to quit compared with Android users [57]. Third, this trial relied on self-reported smoking cessation to estimate 30-day point prevalence abstinence. While expert consensus suggests that biochemical verification of abstinence is impractical and unnecessary in studies similar to this one [58], future research will need to address this by implementing biochemical verification of smoking cessation (eg, exhaled carbon monoxide). Fourth, in a study of an intervention conducted by the developer of the intervention, the potential for biases (eg, rater biases, response biases) are noted. Despite efforts to eliminate the opportunity for such biases through the design and implementation of rigorous study procedures, the potential for bias will limit the extent to which our results can be interpreted beyond being preliminary indicators. Fifth, some users (29 at baseline and 66 by study outcome) were using a pharmacotherapy cessation aid during the trial, which could have contributed to the cessation rates observed and limits the ability to attribute cessation solely to Clickotine. To overcome such limitations, a pivotal study of Clickotine should evaluate efficacy and test superiority in a blinded, randomized controlled trial conducted by an independent party; include iPhone and Android versions of the app; include an active comparator arm with an alternative mobile app; feature longer-term follow-up; and ascertain abstinence via biochemical verification.

In summary, the results of this initial evaluation suggest that Clickotine participants engaged with the app and appeared to remain engaged with the app for a majority of the study duration on average, and that Clickotine use may be associated with cessation outcomes. Future research is warranted to evaluate the engagement with and efficacy of Clickotine in more robust clinical trials, and to assess Clickotine’s long-term efficacy and safety.

## Abbreviations

IRB: institutional review board
ITT: intention-to-treat
USCPG: US clinical practice guideline
